# Artesunate: A Review of Its Potential Therapeutic Effects and Mechanisms in Digestive Diseases

**DOI:** 10.3390/pharmaceutics17030299

**Published:** 2025-02-25

**Authors:** Mengting Shi, Guanhua Ma, Xiulan Yang

**Affiliations:** Department of Pharmacology, The School of Basic Medicine, Health Science Center, Yangtze University, Jingzhou 434023, China; 202106781@yangtzeu.edu.cn (M.S.);

**Keywords:** artesunate, digestive system diseases, pharmacology, inflammation, neoplasm

## Abstract

Artesunate (ART), an artemisinin-derived semi-synthetic sesquiterpene lactone distinguished by its unique endoperoxide group, has become a cornerstone of clinical antimalarial therapy. Recent research has demonstrated its broad pharmacological profile, including its potent antimalarial, anti-inflammatory, anti-tumor, antidiabetic, immunomodulatory, and anti-fibrotic properties. These discoveries have significantly broadened the therapeutic scope of ART and offer new perspectives for its potential use in treating gastrointestinal disorders. Mechanistically, ART exerts significant therapeutic effects against diverse gastrointestinal pathologies—such as gastric ulcers, ulcerative colitis (UC), hepatic fibrosis (HF), gastric cancer, hepatocellular carcinoma, and colorectal cancer—via multimodal mechanisms, including cell cycle modulation, apoptosis induction, the suppression of tumor cell invasion and migration, proliferation inhibition, ferroptosis activation, and immune regulation. This review evaluates existing evidence on ART’s therapeutic applications and molecular mechanisms in digestive diseases, intending to elucidate its clinical translation potential. ART emerges as a promising multi-target agent with significant prospects for improving the management of gastrointestinal disorders.

## 1. Introduction

Approximately one-third of all global prevalence rates are caused by digestive diseases, which contribute to a significant portion of the overall disease burden. Statistics indicate that no discernible downward trend in the prevalence or incidence of digestive diseases worldwide has been observed during the past three decades [1]. Whether it is inflammation or a tumor in the digestive system, both lead to a significant loss of disability-adjusted life years, making them a major component of the global disease burden [2,3]. Digestive diseases are characterized by high morbidity and mortality and are mainly treated with surgery and medicine, with medication serving as the mainstay. Although treatment with Western medicine has a short duration and rapid results, it is possible to develop drug resistance and cause some harm to normal liver and kidney function, which does not entirely satisfy clinical requirements. Chinese medicine therapy offers the benefits of fewer negative effects and better safety, and a wealth of practical and theoretical expertise has been gathered in the treatment of digestive diseases, providing clinical patients with more treatment alternatives [4,5,6,7,8,9,10]. A range of Chinese medicines have positive effects on the treatment of digestive diseases [11,12,13,14], underscoring the necessity for a further exploration of Chinese medicines as therapeutic options.

The artemisinin-derived compound artesunate (ART) is a semi-synthetic agent isolated from *Artemisia annua* L. (Asteraceae), serving as a prodrug of dihydroartemisinin. Its hydrophilic group confers ART with advantages that render it a more effective medication. In comparison to artemisinin, ART demonstrates improved water solubility, significant pharmacokinetic properties, and oral bioavailability. The oral bioavailability of ART is 61%, significantly higher than that of artemisinin, which is approximately 32% [15]. With rapid pharmacodynamic onset, an excellent safety profile, negligible toxicity, and a reduced likelihood of inducing drug resistance, ART has been extensively employed against diverse clinical conditions. In addition to its well-characterized antimalarial action, ART demonstrates broad-spectrum therapeutic effects, encompassing anti-atherosclerotic, anti-inflammatory, antiviral, antineoplastic, antimicrobial, and anti-fibrotic properties [16]. Recent decades have witnessed a growing body of evidence elucidating ART’s therapeutic potential for digestive diseases [17,18,19,20], establishing it as a key investigational target in modern gastroenterological research.

This review aims to provide a theoretical foundation for the use of ART as a clinical drug to treat digestive diseases. The proven and potentially therapeutic roles and mechanisms of ART in various digestive diseases, including gastric ulcers, ulcerative colitis, hepatic fibrosis, gastric cancer, colorectal cancer, hepatocellular carcinoma, etc., are summarized through multiple pathways in different cells.

## 2. The Chemical Structure of ART and Its Biological Activity

**Figure 1:**
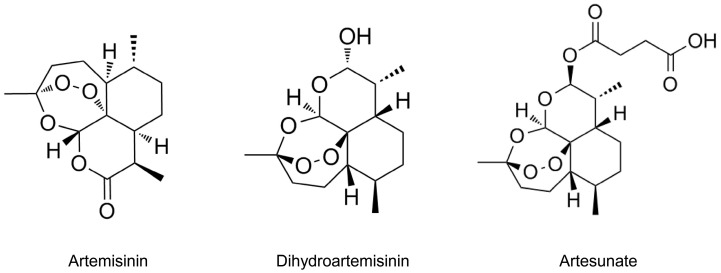
The chemical structures of artemisinin, dihydroartemisinin (DHA), and artesunate (ART).

Artemisinin (see Figure 1) is a sesquiterpene lactone peroxide extracted from the plant *Artemisia annua* L., and it is a primary therapeutic agent for malaria. Despite its efficacy, artemisinin has limitations, including poor water solubility and low bioavailability. To overcome these challenges, researchers have developed derivatives such as ART and dihydroartemisinin (DHA) through structural modifications. One such derivative, ART [21], also known as dihydroartemisinin-12α-succinate, retains the sesquiterpene lactone backbone of artemisinin while significantly enhancing its water solubility and stability through targeted chemical modifications. The molecular formula of ART is C_19_H_28_O_8_, with a molecular weight of 384.42 g/mol. ART maintains the peroxide bridge of artemisinin, a crucial component for its antimalarial activity. By introducing a succinate monoester group at the 12th hydroxyl position through esterification, the water solubility of ART is enhanced. The synthetic pathway from artemisinin to ART comprises two sequential steps: (1) reduction using diisobutylaluminum hydride followed by (2) esterification with succinic anhydride [22].

ART [15] is a weakly acidic compound with a pKa range of 3.5 to 5.5. It demonstrates low ionization in acidic environments and is completely soluble in weakly alkaline solutions, enabling its formulation into various clinical preparations such as injections, tablets, suppositories, and creams for clinical use. Whether administered orally, intravenously, intramuscularly, or rectally, ART is metabolized into DHA in the body. The excretion of DHA is catalyzed by UDP-glucuronosyltransferases to α-DHA-β-glucuronide, which is ultimately excreted in urine [23].

After the intravenous administration of ART, the drug is rapidly metabolized within the body by enzymatic hydrolysis into DHA. Studies indicate that the average half-life of ART is less than 15 min. In patients with severe malaria, following an initial intravenous dose of 2.4 g/L of ART, the peak concentration (C_max_) ranges around 110 (13 to 520) nmol/L, with an elimination half-life (t_1/2_) of 13.2 (4.8 to 36.6) min [24]. ART is eliminated from the body at a first-order linear rate when administered intravenously at doses ranging from 0.5 to 8 mg/kg [25]. The average clearance rate of ART is 2 to 3 L/kg/h. Notably, the C_max_ of ART achieved with intravenous administration is significantly higher than that observed with other routes of administration. In contrast, ART administered intramuscularly is absorbed more rapidly and reaches its C_max_ sooner. The absolute bioavailability of ART, calculated from the concentration of the active metabolite DHA, is 86.4% in adults [26] and 88% in children [27] infected with severe malaria. The average time to maximum concentration (T_max_) following intramuscular administration is 7.2 to 12 min, with an average half-life of ART of 25.2 to 48.2 min. Compared to intravenous administration, muscle injection leads to a lower C_max_ and a longer t_1/2_ of ART. The oral administration of ART results in the drug being primarily absorbed in the small intestine. ART can be detected in the bloodstream within 15 min post-administration, reaching its C_max_ after one hour, and is eliminated within a half-life period of 20 to 45 min. The absolute bioavailability of oral ART is 61% [28]. A portion of ART is absorbed into the bloodstream and converted to DHA, with a bioavailability of 80% to 85% [24], while another portion enters the liver via the portal vein, where it is metabolized into DHA. Regarding rectal administration, the T_max_ for ART ranges from 34.8 to 85.8 min [29,30]. The pharmacokinetic parameters are generally similar to those of oral administration, although the T_max_ and half-life of ART following rectal administration are significantly higher than those observed with oral administration.

Cytochrome P450 (CYP450) enzymes are pivotal in the process of drug metabolism and play a crucial role in mediating drug interactions [31]. The pharmacokinetic properties of ART are intricately associated with its modulation of the CYP450 enzyme system. Research has demonstrated that ART can reversibly inhibit the enzyme activities of CYP2C19 and CYP3A4, potentially altering the metabolic clearance rates of drugs when co-administered [32]. Furthermore, prolonged exposure to ART may lead to the upregulation of the gene expression for CYP3A4 and CYP2B6 [33]. This bidirectional regulation of the CYP450 enzyme system by ART can result in complex drug interactions. Consequently, in clinical practice, it is imperative to integrate population pharmacokinetic models with genomic data to refine the dosing regimens for ART when it is used in conjunction with other therapeutic agents.

ART is widely recognized by the public as a highly effective antimalarial drug and is extensively used in clinical malaria treatment [34]. In addition to its antimalarial properties, numerous studies have revealed that ART also possesses a range of pharmacological effects. It can exhibit anti-tumor activity by regulating the cell cycle, inducing apoptosis, inhibiting invasion and migration, and suppressing proliferation [35,36,37,38,39]; alleviate inflammation by engaging apoptotic signaling, mediating immune regulation, and reducing oxidative stress [40,41,42]; enhance the sensitivity of common clinical drug-resistant bacteria to antibiotics [43,44]; demonstrate antiviral effects by inhibiting the replication of various viral families [45,46,47]; exert anti-parasitic effects by targeting mitochondria and cell membranes and inhibiting the growth of parasitic worms [48,49,50]; and display anti-fibrotic properties by suppressing fibroblast proliferation, promoting apoptosis, and regulating various cytokines, among others [51,52,53,54].

All of the aforementioned pathophysiologic processes may be implicated in a range of digestive diseases, suggesting a potential role for ART in the prevention and treatment of certain digestive diseases, a hypothesis that has been explored and demonstrated in several studies.

## 3. The Therapeutic Advantages of ART

### 3.1. High Safety

In a clinical trial, twenty-four healthy volunteers were administered daily doses of 2, 4, and 8 mg/kg via intravenous injection within 2 min. After a consecutive 3-day regimen, the tolerance to the intravenous administration of ART was favorable. The volunteers exhibited no serious adverse effects, no decline in drug concentration was observed, and no accumulation was detected, suggesting that the 8 mg/kg intravenous administration at a high dose was safe [25]. Additionally, in a randomized controlled trial involving 5425 children diagnosed with severe malaria, 2712 patients were administered ART at a dosage of 2.4 mg/kg every 12 and 24 h post-admission, whereas 2713 patients were treated with quinine. The findings revealed that ART significantly decreased the mortality rate in children with severe malaria and was accompanied by a reduced incidence of neurological sequelae and hypoglycemia relative to the quinine group, suggesting that ART exhibits a favorable safety profile with no notable drug-related adverse effects [55]. Current studies indicate that ART demonstrates efficacy in treating malaria during pregnancy with a favorable tolerance profile. Excluding those lost to follow-up, the remaining women experienced uncomplicated deliveries. Of the newborns, 29% exhibited low birth weight, with no congenital anomalies identified. Following a one-year follow-up period, all infants were found to be at normal developmental milestones. The findings indicate that the administration of ART does not elevate the risk of adverse pregnancy outcomes and poses no harmful effects on infant health [56].

Extensive research has demonstrated that ART exhibits a high degree of safety and is well tolerated in clinical settings, with no serious adverse reactions reported in children or pregnant women. Consequently, artemether-lumefantrine is considered safe for clinical use.

### 3.2. Less Toxic Side Effects

No severe adverse reactions were observed among patients administered ART, with only mild symptoms such as slight fever, fatigue, leukopenia, anemia, dizziness, nausea, vomiting, neutropenia, and rashes reported. Furthermore, these adverse events do not pose a life-threatening risk [57,58,59]. In a study investigating the ototoxic effects of ART use, 23 patients with advanced breast cancer were treated with the drug at a dose of 0, 100, or 200 mg daily for a continuous four-week period as an adjunct to tumor therapy. The findings revealed that participants demonstrated good tolerance to the drug with respect to both the auditory and vestibular systems. During treatment, four instances of mild auditory system impairment were observed, which resolved without the need for additional medication. Additionally, four cases of vestibular system side effects were reported, and they fully resolved following the discontinuation of the medication [60]. Among patients treated with ART, no instances of drug-related brainstem toxicity or neurophysiologic adverse events were detected [61].

In the current clinical trials, no significant impairment in liver and kidney function or alterations in blood biochemistry parameters have been reported with the use of ART. The toxicity of ART is caused by sustained exposure to therapeutic concentrations, as opposed to short-term peak levels. Consequently, the vigilant monitoring of patients’ responses to the drug is imperative throughout the duration of long-term therapy [62].

### 3.3. Reverse Tumor Drug Resistance

Tumors often develop resistance to conventional chemotherapeutic agents, yet they retain a high level of sensitivity to ART, making ART a promising candidate for reversing resistance in tumors that have become resistant to standard chemotherapies. Research has demonstrated that ART exhibits significant inhibitory effects on cancer cells with increased lysosomal function-induced resistance, including human lung adenocarcinoma cells resistant to cisplatin (A549/DDP), human breast cancer cells resistant to doxorubicin (MCF-7/ADM), and human lung adenocarcinoma cells resistant to paclitaxel (A549/PTX) [63]. In acute myeloid leukemia cells that are resistant to cytarabine, ART can enhance sensitivity to cytarabine by modulating the Janus kinase/signal transducer and activator of the transcription JAK/STAT3 pathway [64]. Multidrug resistance in tumor cells is associated with the overexpression of ATP-binding cassette (ABC) transporters, and ART can reverse tumor resistance by downregulating the expression of ATP-binding cassette subfamily G member 2 (ABCG2) [65,66].

## 4. Effects and Mechanisms of ART

**Figure 2:**
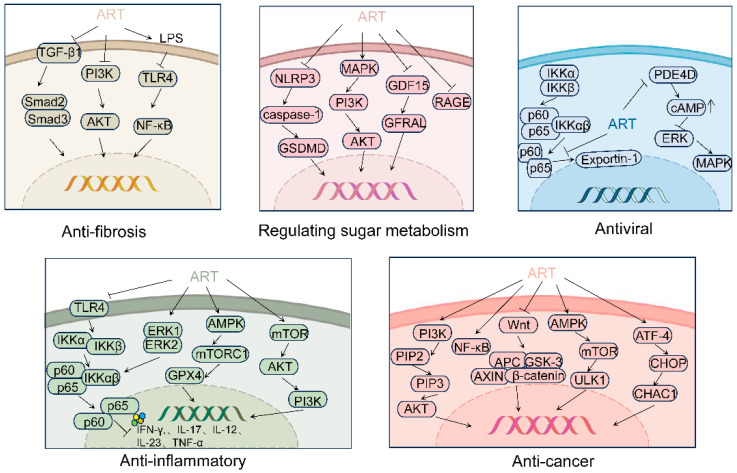
Molecular mechanism of ART. ART can regulate a wide range of molecules and signaling pathways to exert anti-inflammatory, anti-cancer, anti-fibrotic, antiviral, and metabolic regulatory effects on sugar metabolism. (“→” indicates promotion; “⊥” indicates inhibition; “↑” indicates upregulation).

Numerous studies indicate that ART can play a significant role in the anti-inflammatory [67], anti-fibrotic [68], anti-cancer [69], antiviral [70], and metabolic regulation of sugar properties [71]. ART can be employed for the treatment of disease by inhibiting inflammatory factors, alleviating oxidative stress, modulating the cell cycle, and inhibiting proliferation, processes that are connected with several signaling pathways (see Figure 2).

In investigations of diverse inflammatory conditions, Nuclear factor kappa B (NF-κB) activation acts as a pivotal transcription factor in the initiation of inflammation [72]. Research indicates that ART effectively downregulates the expression of pro-inflammatory cytokines, including Tumor necrosis factor-α (TNF-α) and Interleukin (IL)-1β, thereby inhibiting the NF-κB pathway and mitigating inflammation [73]. ART suppresses the TLR4/NF-κB signaling pathway, enhancing inflammation control and exerting a protective function by diminishing the expression of pro-inflammatory cytokines including Interferon (IFN)-γ, IL-17, IL-12, IL-23, and TNF-α [74,75,76,77]. Moreover, ART inhibits the ERK1/2/NF-κB/IL-1β pathway by downregulating NLRP3 expression, thereby exerting anti-inflammatory effects [78]. ART exerts neuroprotective effects by inhibiting inflammation through the AMPK/mTORC1/GPX4 pathway [41]. Additionally, ART modulates the mTOR/AKT/PI3K pathway, thereby defending against LPS-induced acute lung injury [79].

Research on various cancers suggests that cancer development is predominantly driven by genetic mutations and the dysregulation of gene expression [80]. These mutations can lead to the activation of oncogenes and the inactivation of tumor suppressor genes, thereby impacting critical biological processes, including cell cycle regulation, DNA repair, and apoptosis [81]. Additionally, processes including inflammation, immune evasion, and epigenetic changes also contribute significantly to the initiation and progression of cancer [82]. ART can inhibit tumor cell proliferation and migration through the modulation of the PI3K/AKT signaling pathway [83,84,85]. Extensive research has confirmed that tumor development mechanisms are intimately linked with the activation of the NF-κB signaling pathway [86,87,88]. Multiple studies have shown that ART can inhibit the NF-κB pathway at multiple levels, thereby exerting anti-cancer effects [86]. Furthermore, ART can suppress cancer cell proliferation by inhibiting the transduction of the Wnt/β-catenin signaling pathway [89,90,91]. Additionally, ART can induce apoptosis in cancer cells via the ATF4/CHOP/CHAC1 pathway [92]. ART triggers autophagy-dependent apoptosis by activating the AMPK/mTOR/ULK1 pathway [38].

Research on various fibrotic diseases reveals that Transforming growth factor (TGF)-β1 plays a pivotal role in the fibrotic process, with the TGF-β1/Smad2/3 pathway serving as a crucial anti-fibrotic mechanism. ART can improve fibrosis by downregulating the expression of TGF-β1 and Smad3 [93]. Furthermore, ART can reduce fibroblast activation and decrease GPX4 expression, thereby inducing mitochondria-dependent iron death by inhibiting the TGF-β1/Smad2/3 and PI3K/AKT pathways, which in turn improves fibrosis [94]. Additionally, ART exerts anti-fibrotic effects by mitigating inflammatory responses through the LPS/TLR4/NF-κB signaling pathway [51].

Research into various viral diseases indicates that the invasion of viruses into host cells and the use of these cells for replication are the primary reasons for the onset of viral diseases [95]. ART can inhibit the NF-κB pathway by blocking the interaction between NF-κB p65 and Exportin-1, which recognizes the nuclear export of p65, thus preventing p65 from exiting the nucleus and reducing the activation of p65 in the cytoplasm, thereby inhibiting the replication of herpesviruses within the body [96]. Furthermore, ART can directly inhibit recombinant Phosphodiesterase 4D (PDE4D), leading to the accumulation of cAMP within the cell, which in turn blocks the ERK/MAPK pathway, ultimately inhibiting the replication of influenza A virus [97].

The occurrence of diseases related to glucose metabolism is associated with genetic mutations leading to the loss of or alterations in key enzyme functions [98], mutations or dysfunction in the insulin signaling pathway [99], mitochondrial dysfunction [100], and the impact of oxidative stress on glycolysis and the tricarboxylic acid cycle [101]. ART can regulate blood glucose levels and protect pancreatic β-cells through the modulation of the NLRP3/caspase-1/GSDMD pathway [102]. ART can modulate the MAPK/PI3K/AKT signaling pathway, thereby improving metabolic disorders [103]. Moreover, ART can mitigate metabolic abnormalities related to obesity through the inhibition of the GDF15/GFRAL signaling pathway [104]. At high concentrations, ART effectively suppresses RAGE expression, leading to a reduction in blood glucose levels [105].

## 5. ART and Digestive System Diseases

### 5.1. Gastric Ulcer

Gastric ulcer is a prevalent digestive disease, impacting more than 10,000 people worldwide annually, and its severity may cause gastric hemorrhage, gastric perforation, and even cancerous tendencies [106]. Currently, Western medicines, including proton pump inhibitors, histamine receptor inhibitors, and H2 receptor antagonists, are commonly used in clinical settings as treatment options. However, the prolonged administration of these drugs can frequently lead to various adverse reactions such as allergies and hematopoietic dysfunction. In recent years, research has demonstrated that traditional Chinese medicine has significant advantages in treating gastric ulcers. ART can effectively reduce damage to the gastric mucosa, inhibit gastric acid secretion, and suppress the activity of *Helicobacter pylori*, thereby exerting a therapeutic effect on gastric ulcers [107].

Oxidative stress and the release of inflammatory factors cause damage to the gastric mucosa, which are important mechanisms in the development of gastric ulcers. In a rat model of gastric ulcers induced by ethanol induction and pyloric ligation [108], as well as by aspirin [109], ART significantly reduced gastric mucosal damage, inhibited gastric acid secretion, increased the pH of gastric secretions, and significantly decreased Glutathione (GSH) and Superoxide dismutase (SOD) activity; TNF-α levels; and the level of inflammatory mediators IL-1β, IL-6, and NF-κB (p65) while elevating Thiobarbituric acid reactive substances (TBARS) and Myeloperoxidase (MPO) levels. Furthermore, ART at dosages of 50 and 150 mg/kg has a neutralizing effect on secretions and exerts a therapeutic effect on gastric ulcers by maintaining oxidative homeostasis and downregulating the expression of inflammatory factors.

**Figure 3:**
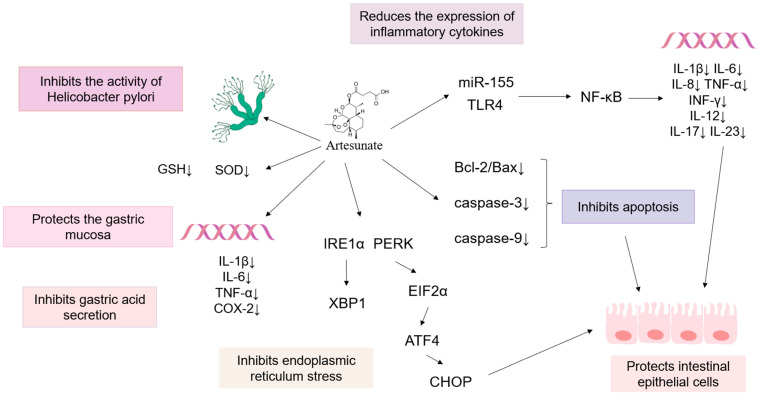
The mechanism of ART in the treatment of the inflammation of the digestive system. ART can treat gastric ulcers and ulcerative colitis through a variety of anti-inflammatory mechanisms. (“↓” indicates downregulation).

*Helicobacter pylori* (*H. pylori*) infection and the use of nonsteroidal anti-inflammatory drugs are primary contributors to the pathogenesis of gastric ulcers. Research studies have demonstrated that the artesunate dry emulsion formulation (ADEF) effectively inhibits the growth of a diverse range of *H. pylori* and has significant in vitro antimicrobial activity. In vivo experiments in mice infected with the *H. pylori* PremSS1 strain demonstrated that the combination of ADEF with amoxicillin and PPIs led to a 90% eradication rate of *H. pylori*. ADEF can serve as an alternative to antibiotics for treating *H. pylori* dry infections and *H. pylori*-caused gastric ulcers [110] (see Figure 3 and Table 1).

### 5.2. Ulcerative Colitis

Ulcerative colitis (UC) is a nonspecific, recurrence inflammatory disorder of the colonic mucosa affecting the rectum and colon. Damage to the intestinal barrier leads to the displacement of gut microbiota and the overactivation of the immune system, which results in inflammatory reactions [111]. No complete cure for UC is available in clinical practice, and treatments such as aminosalicylic acid preparations, immune or biological inhibitors, and adrenal glucocorticoids are commonly employed. However, these therapies necessitate long-term maintenance treatment and are susceptible to recurrence. The long-term use of these drugs has adverse effects on hematopoietic, liver, kidney, and other functions, and individual responses to drug treatment vary significantly, resulting in unstable effects. Current research indicates that ART can reduce the apoptosis of normal cells, inhibit endoplasmic reticulum stress, exert protective effects on colon tissue through antioxidant stress, and exhibit a lower incidence of adverse effects when compared to conventional Western medicine [112].

For the dextran sulfate sodium (DSS)-induced UC model, ART preserves the intestinal barrier through the maintenance of mucin 2 (Muc2) and Claudin-1 expression in intestinal epithelial cells, the regulation of the B-cell lymphoma-2/Bcl-2-associated X protein (Bcl-2/Bax) ratio, and the reduction in cleaved-caspase-3 expression in the colon tissue. Additionally, ART potently inhibits the phosphorylation of NF-κB p65 and the Inhibitor of NF-κB α (IκBα) and suppresses the mRNA expression of IL-1β, IL-6, and TNF-α. These findings suggest that ART exhibits preventive and protective properties against UC in mice, preserving the integrity of the intestinal barrier and dampening inflammatory responses [113]. In the in vitro UC model constructed using LPS-induced RAW264.7 cells [114], ART substantially reduced MicroRNA 155 (miR-155) levels in LPS-induced RAW264.7 cells in a concentration-dependent fashion; improved cell survival; and lowered the expression levels of pro-inflammatory cytokines including IL-12, IL-17, IL-23, and TNF-α. Additionally, in vivo experiments conducted in the Trinitro-benzene-sulfonic acid (TNBS)-induced murine UC model showed that ART suppressed the activation of the NF-κB signaling pathway, with results consistent with those from in vitro experiments, indicating that ART can modulate the miR-155/NF-κB signaling pathway to inhibit inflammatory effects. Similarly, Chen et al. [115] used LPS-induced RAW264.7 cells to construct a UC model for in vitro experiments and a DSS-induced UC rat model for in vivo experiments. The results from the in vivo and in vitro experiments demonstrated that ART could reduce MPO activity to enhance the antioxidative stress ability of UC rats and afford protection to the colon while inhibiting TLR4, p-NF-κB, Bax, caspase-9, TNF-α, IL-8, and IFN-γ. ART inhibits signaling activity within the TLR4/NF-κB signaling pathway, thereby achieving a therapeutic effect.

Studies have demonstrated that ART could alleviate endoplasmic reticulum stress in mouse colon tissues by inhibiting the DSS-induced upregulation of proteins associated with the endoplasmic reticulum stress response, specifically Glucose-regulated protein 78Kd (GRP78) and C/EBP homologous protein (CHOP); moreover, ART may decrease endoplasmic reticulum stress in mouse colon tissue by modulating the p-PERK-eIF2α-ATF4-CHOP and IRE1α-XBP1 signaling pathways, specifically through the suppression of p-PERK/PERK, p-eIF2α/eIF2α, p-IRE1α/IRE1α, activating transcription factor 4 (ATF4), and X-Box Binding Protein 1 (XBP1) protein levels [116] (see Figure 3 and Table 2).

### 5.3. Hepatic Fibrosis

Hepatic fibrosis (HF) is defined by the accumulation of extracellular matrix (ECM) and the formation of scarring, which is a wound-healing reaction to the sustained action of multiple injury factors [117]. At present, hepatic fibrosis is mainly treated with etiological treatment and anti-fibrotic treatment, such as the use of drugs like interferon and corticosteroids. Etiological treatment using Western medicine can help inhibit or even reverse hepatic fibrosis, although its effectiveness in treating fibrosis based on the etiology remains limited, and it cannot fully inhibit inflammation. In the past decade, with the continuous development of traditional Chinese medicine, the therapeutic effectiveness and safety of traditional Chinese medicine for hepatic fibrosis have been substantiated by extensive research, and Chinese medicine has broad application prospects [118]. ART, as an extract of artemisinin, can exert anti-fibrotic effects through the inhibition of hepatic stellate cell (HSC) activation, the induction of autophagy and apoptosis in HSCs, and the suppression of collagen production.

In the HF model established in mice by carbon tetrachloride induction, ART effectively suppressed the expression of ferroptosis markers GPX4 and Prostaglandin-endoperoxide synthase 2 (Ptgs2) in the fibrotic liver tissues of mice. To elucidate the cells undergoing ferroptosis, primary HSCs were isolated from mouse livers, and the results were consistent with those observed in vivo: ART inhibited cell viability and induced intracellular Fe^2+^ accumulation; led to an excess of Lipid hydroperoxide (LPO) and Malondialdehyde (MDA); and caused the deletion of reactive oxygen species (ROS)-scavenging enzymes GSH, GPX4, and Nicotinamide adenine dinucleotide phosphate (NADPH), resulting in ferroptosis within activated HSCs. Similarly, LX2 human liver stellate cell lines were treated with ART at different concentrations, and in vitro experiments showed that the LX-2 cells treated with ART exhibited the expression of ferritin autophagy-related genes, including autophagy-related gene 3 (*Atg3*), *Atg5*, *Atg6*/*beclin1*, and *Atg12*; the levels of Microtubule-associated protein 1 light chain 3 (LC3) were significantly elevated; and the levels of p62, ferritin heavy chain 1 (FTH1), and Nuclear receptor coactivator 4 (NCOA4) were decreased. ART may induce ferroptosis in activated HSCs by activating ferritin autophagy, which may exert anti-fibrotic effects [119].

The HSC secretion of the ECM is dependent on the energy provided by mitochondria. For HF induced by schistosome infection, ART decreases the mitochondrial oxygen consumption rate (OCR) and reduces the protein expression of the NDUFB8 subunit of complex I and the UQCRC2 subunit of complex III in HSCs. ART may alleviate HF by inhibiting mitochondrial function in HSCs [120].

Furthermore, Xu et al. [121] discovered that in a rat HF model induced by bovine serum albumin, ART suppressed the expression of hydroxyproline, Alpha smooth muscle Actin (α-SMA), and type I collagen; reduced the expression of matrix metalloproteinase-2 (MMP-2) and MMP-9 while increasing MMP-13 expression; and decreased collagen content, leading to a marked improvement in HF in mice. The mechanism of the ART inhibition of HF may be related to the downregulation of MMP-2 and MMP-9, coupled with the upsurge in MMP-13 levels.

In vitro, human HSC line LX-2 cells were cultured, and it was found that ART inhibited proliferation and dose-dependently elevated the Bax/Bcl-2 ratio and promoted apoptosis in LX-2 cells. These cells can downregulate the levels of α-SMA and Collagen Type I (COL1) and prevent the activation of LX-2 cells. Furthermore, ART suppressed β-catenin expression and the phosphorylation of Glycogen Synthase Kinase-3β (GSK-3β), AKT, and Focal Adhesion Kinase (FAK). ART may inhibit the development of HF by inhibiting the FAK/AKT/β-catenin pathway [122].

In a carbon tetrachloride-induced animal model of HF, ART could inhibit the upregulation of TLR4, α-SMA, TGF-β1, and Myeloid Differentiation Factor (MyD88) at both the protein and mRNA levels and reduce the production of pro-inflammatory factors TNF-α and IL-6, as well as significantly inhibiting the levels of NF-κB p65. These results suggested that ART may be able to alleviate HF and inflammation through its suppression of the LPS/TLR4/NF-κB signaling pathway, effectively inhibiting the activation of HSCs [123].

**Figure 4:**
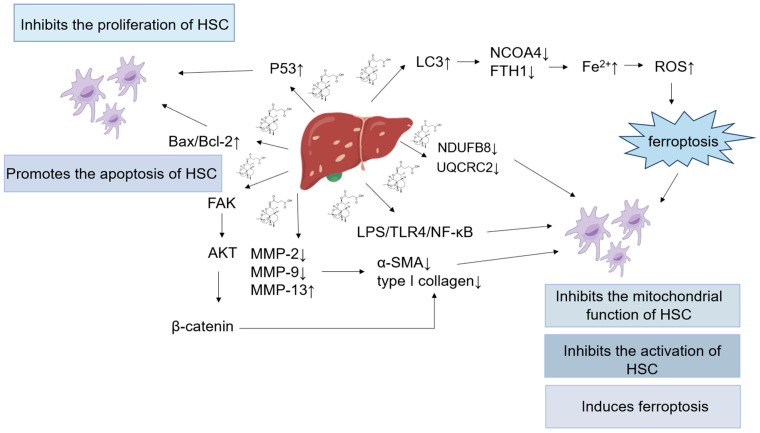
Mechanism of action of ART in treatment of hepatic fibrosis. ART can exert anti-fibrotic effects through the inhibition of HSC activation, the induction of autophagy and apoptosis in HSCs, and the suppression of collagen production. (“↑” indicates upregulation; “↓” indicates downregulation).

Longxi et al. [124] isolated HSCs from rats for in vitro experiments, and ART suppressed cell proliferation and induced apoptosis while decreasing hydroxyproline levels and reducing collagen production. Additionally, ART increased the levels of p53 mRNA and p53 protein. ART suppresses the proliferation of primary HSC through the upregulation of p53 expression, which leads to G1 phase arrest and apoptosis, and exerts anti-fibrotic effects (see Figure 4 and Table 3).

### 5.4. Gastric Cancer

Gastric cancer is the fifth most prevalent cancer worldwide. It is among the most lethal cancers affecting the general population, accounting for the third highest incidence of malignant tumors in China [125]. Currently, surgical intervention, chemotherapy, radiotherapy, targeted therapy, and immunotherapy are employed as treatment modalities for gastric cancer [126]. However, treatment with conventional Western medicine for advanced gastric cancer is associated with limited efficacy and substantial adverse effects and significantly impairs patients’ quality of life [127]. As an effective treatment for gastric cancer, traditional Chinese medicine has unique advantages across various stages of gastric cancer’s occurrence and development. Chinese medicine is capable of alleviating clinical symptoms, enhancing life satisfaction, and prolonging the survival duration of patients [128]. ART can exert anti-cancer effects through its ability to promote apoptosis in gastric cancer cells and suppress their proliferation.

Wang et al. [129] demonstrated that ART exhibits inhibitory effects on the proliferation of SGC-7901 cells and induces apoptosis in them. The mechanism for this might be related to the fact that ART significantly downregulates cell division cycle 25 homolog A (CDC25A) and Bcl-2 protein expression, promoting the levels of Bax and caspase-3, along with decreasing the mitochondrial membrane potential. Additionally, ART can induce apoptosis in HGC27 cells by suppressing COX-2 expression, leading to a decline in Bcl-2 levels, the upregulation of Bax, and the promotion of caspase-3 and caspase-9 expression [130].

In vitro (SGC7901, BGC-823, and AGS cells) and in vivo (nude mouse models) studies demonstrated that calcium overload occurred, Vascular Endothelial Growth Factor (VEGF) expression was decreased, and calpain-2 expression was elevated in ART-treated BGC-823 cells. The mechanism of its action may be that ART exhibits a concentration-dependent inhibition of gastric cancer in vitro and in vivo by affecting the expression of calcium, VEGF, and calpain-2 [131].

**Figure 5:**
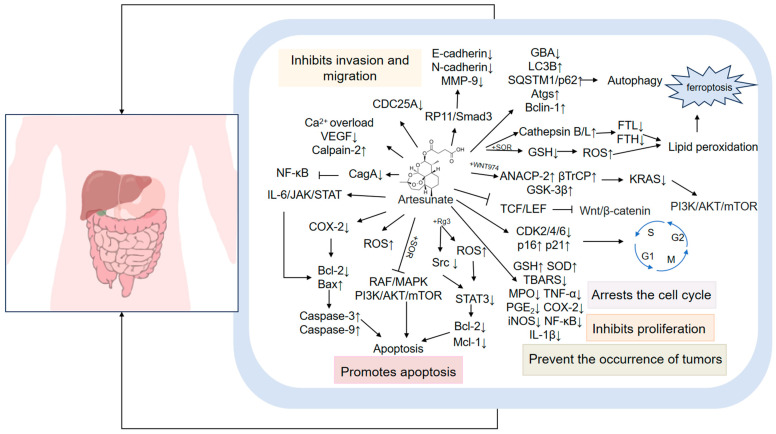
Mechanism of action of ART in treatment of gastric cancer, hepatocellular carcinoma, and colorectal cancer. ART can exert anti-cancer effects by inhibiting tumor cell proliferation, promoting apoptosis, inhibiting invasion and migration, and inducing ferroptosis. (“→” indicates promotion; “⊥” indicates inhibition;”↑” indicates upregulation; “↓” indicates downregulation).

Su et al. [132] noted that for gastric cancer caused by *H. pylori*, ART inhibited the proliferation of *H. pylori* and cells, suppressed the adhesion of *H. pylori* to various gastric cancer cell lines (SGC-7901 and GES-1 cells), and diminished ROS production induced by *H. pylori*. Moreover, ART increased IκBα expression while suppressing COX-2 and p-IκBα expression. The mechanism of action involved the inhibition of NF-κB activation (see Figure 5 and Table 4).

### 5.5. Hepatocellular Carcinoma

Primary hepatocellular carcinoma [133,134,135] originates in hepatocytes or intrahepatic bile duct cells, ranking as the fifth most prevalent form of malignant tumor in China. Most hepatocellular carcinoma cases are diagnosed at advanced stages, and surgical treatment is ineffective. Therefore, transcatheter arterial chemoembolization (TACE), immunotherapy, targeted therapy, and combination therapy have emerged as the primary modalities for managing intermediate and advanced hepatocellular carcinoma. However, these modalities may cause varying degrees of impairment to bodily function and are associated with significant costs. Targeted therapy has certain drug resistance and a range of adverse reactions, while immunotherapy has a lengthy treatment duration and slow effectiveness. Many traditional Chinese remedies have been proven to offer unique advantages and good clinical efficacy in the management of hepatocellular carcinoma through in-depth research. ART can impede the growth, invasion, and migration of HCC cells, as well as inducing ferroptosis and apoptosis in such cells, and its combined effects with sorafenib are more significant.

Jing et al. [136] cultured SK-HEP1, SM7721, HepG2, and Huh7 cells in vitro, which showed that ART could inhibit EMT by downregulating the levels of E-cadherin, N-cadherin, MMP-9, and Smad2/3, thereby mitigating the invasive and migratory behavior of HCC cells, whereas the overexpression of RP11 could reverse the effects of ART. ART inhibits EMT by inhibiting the RP11/Smad3 signaling pathway, thus exerting anti-hepatocellular carcinoma effects.

In vitro (HepG2, SNU-182, SNU-449, Huh7 cells) and in vivo (nude mice inoculated with Huh7 cells) research demonstrated that ART enhanced the sensitivity of HCC to sorafenib and that sorafenib in combination with ART exhibited a synergistic efficacy in the killing of cells. This combination treatment could reduce GSH synthesis and increase ROS production, which could decrease mitochondrial potential and impair the function of mitochondria, and it could induce the activation of Cathepsin B/L and ferritin degradation. Both agents synergistically reduced Vascular Endothelial Growth Factor (TFRC) expression and the concomitant degradation of Ferritin light chain (FTL) and ferritin heavy chain (FTH), thus promoting HCC cells toward ferroptosis [137].

In vitro experiments with HCC cell lines (PLC/PRF/5, HuH7, HepG2, Hep3B, and HCCLM3 cells) showed that ART administration markedly augmented the concentrations of p-ERK and p-STAT3 and was able to induce increased levels of ROS, which led to the suppression of Bcl-2 expression, an enhancement in Bax expression, a rise in caspase-3/7 activity, and cell apoptosis. In vivo (nude mice inoculated with Hep3B cells), the combination of artesunate and sorafenib effectively inhibited tumor growth. The combination treatment of sorafenib and ART exhibits a synergistic effect in inhibiting the growth of HCC cells, as well as in inducing apoptosis, and the mechanism is related to the inhibition of ART’s activation of the ERK and STAT3 signaling pathways by sorafenib [138]. In addition, Ginsenoside Rg3 combined with ART treatment significantly inhibits tumor growth in mice (inoculation of HepG2 SR cells into mice) and suppresses the phosphorylation of STAT3 and its upstream kinase, Src, in HepG2 cells. This leads to a reduction in STAT3 levels and the downregulation of Mcl-1 and Bcl-2 protein levels, thereby promoting apoptosis in tumor cells. The mechanism of action may be associated with the restraint of the Src/STAT3 pathway and the regulation of the ROS/STAT3 pathway [139]. Similarly, in vitro (SK-hep1 and SM-7721 cells) and in vivo (inoculation of SK-hep1 into mice) studies have shown that the combination of sorafenib and ART significantly promoted tumor cell apoptosis through the inhibition of the RAF/MAPK and PI3K/AKT/mTOR pathways, which in turn exerted anti-cancer effects [140]. In the hepatocellular carcinoma model established by nitroso diethylamine, ART markedly suppressed the levels of Alkaline Phosphatase (ALP), Aspartate Aminotransferase (AST), Alanine Aminotransferase (ALT), LDH, and the pre-tumor marker g-GT, as well as decreasing the levels of IL-6, JAK-2, STAT-3 (pY705), Bcl-xL, and Bcl-2 expression and upregulating the levels of caspase-3 expression. ART exerts its antiproliferative and pro-apoptotic effects by inhibiting the IL-6-JAK-STAT pathway [141].

An analysis of clinical hepatocellular carcinoma tissues revealed that the overexpression of GBA was an important factor in the poor prognosis of patients with hepatocellular carcinoma and that there was also a high expression of GBA in HepG2 and MHCC-97H cells. In both in vitro (HepG2 and MHCC-97H cells) and in vivo (a rat model with HepG2 cells) experiments, it was found that ART could promote the apoptosis of tumor cells in a manner that increased with the amount used, whereas a combination with GBA activators reversed the effects of ART; ART also facilitated the transformation of LC3B-I into LC3B-II, increased the levels of SQSTM1/p62, decreased GBA protein levels, and markedly elevated the count of autophagosomes within HCC cells. ART may exert anti-cancer effects by attenuating GBA-mediated autophagy [142] (see Figure 5 and Table 5).

### 5.6. Colorectal Cancer

Colorectal cancer [143,144] is a prevalent form of malignant tumor that affects the digestive tract. It ranks second in terms of incidence rate, following lung cancer. Currently, radical resection remains the most effective treatment modality for colorectal cancer. Besides surgery, radiotherapy, chemotherapy, immunotherapy, and targeted therapy are also commonly used in the treatment of colorectal cancer. Chemotherapy drugs have many toxic side effects and are prone to developing drug resistance, resulting in poor prognosis. Over the past decade, there has been a growing body of scientific research demonstrating that many traditional Chinese medicines have significant effects on the treatment of colorectal cancer. ART can play a protective role by inhibiting the growth of cancer cells, inhibiting inflammatory reactions and oxidative stress, and promoting the apoptosis of tumor cells.

A study by Hamoya et al. [145] demonstrates that ART can inhibit the formation of intestinal polyps in mice. To elucidate the intracellular mechanism underlying this effect, in vitro experiments utilizing DLD-1 and HCT116 cells revealed that ART inhibited the levels of Wnt/β-catenin signaling-related factors, c-Myc, and cyclinD1 proteins and decreased the expression of TCF1/TCF7 in the nucleus. The mechanism for this is that ART inhibits Wnt signaling by binding RNA to inhibit TCF/LEF promoter transcriptional activity, thereby preventing the occurrence of intestinal tumors.

In vitro experiments (HCT116, HT29, SW480, SW620, COLO325, COLO205, HCT15, and RKO cells) revealed that ART combined with WNT974 could reduce the level of KRAS proteins in cells and their activity and upregulate the expression of ANACP-2, βTrCP, and GSK-3β to induce Kirsten rats arcomaviral oncogene homolog (KRAS) protein degradation. Additionally, ART reduced the levels of AKT, PI3K, and mTOR in HCT116 and SW620 cells. In a mouse model with HCT116 cell implants, the combined treatment suppressed tumor growth in mice, consistent with the results from the in vitro experiments. The mechanism for this involves the suppression of the KRAS downstream pathway PI3K/AKT/mTOR by ART to suppress tumor cell proliferation [146].

Z. Huang et al. [147] cultured SW480 and HCT116 cells and treated them with different concentrations of ART and found that ART could damage mitochondria to increase ROS production, decrease the levels of CDK2/4/6, and upregulate the expression of p16 and p21 to block the cell cycle. ART led to an increase in the expression of proteins associated with autophagy, such as LC3B, p62, Beclin1, Atg3, Atg5, Atg7, and Atg12, which induce autophagy. Furthermore, it decreased the expression of BIP and p-IRE1α, thereby inducing endoplasmic reticulum stress. Additionally, ART upregulated the protein levels of IL-6 and MMP-3, which induced senescence in cancer cells. It was shown that ART exerts its inhibitory effect on colorectal cancer growth through the modulation of an intricate interplay involving mitochondrial reactive oxygen species, cellular aging, endoplasmic reticulum strain, and the process of autophagy.

For the 1,2-dimethylhydrazine-induced colorectal cancer rat model, ART resulted in an increase in GSH and SOD concentrations, a reduction in TBARS levels, and the restoration of oxidative stress marker levels to normalcy. Additionally, it led to the suppression of inflammatory markers, such as MPO, TNF-α, PGE2, COX-2, iNOS, NF-κB, and IL-1β. ART mitigates the risk of colon cancer through the suppression of inflammatory and oxidative stress pathways [148].

Jiang et al. [149] cultured HCT116 cells in vitro and inoculated mice with HCT116 cells to establish a colorectal cancer model and treated them with ART. The study revealed that ART suppressed the function of HCT116 cells and triggered cell death by apoptosis; promoted the expression levels of caspase-3, Bax, PARP, and caspase 9 proteins; and decreased Bcl-2 and Bcl-xL expression. ART also increased the levels of autophagy-related proteins such as beclin-1 and LC3-I/II, increased Atg5 levels and Atg12-Atg5 coupling, and promoted cellular autophagy. ART effectively reduces the volume and mass of tumors within the body. The mechanism for this is that ART exerts anti-cancer effects by inducing autophagy and apoptosis in HCT116 cells (see Figure 5 and Table 6).

## 6. Clinical Research Progress of ART Application in Digestive System Diseases

ART is currently a first-line treatment for clinical malaria, widely utilized due to its high safety profile and low toxicity. This has led to its expanded use in clinical research for the management of various other diseases. Extensive clinical research and applications exist for the treatment of malaria and cancer; however, research into ART’s potential use for digestive system diseases is limited [150,151,152,153].

A single-center, randomized, double-blind trial evaluated the anti-cancer efficacy and tolerability of oral ART in patients with colorectal cancer. Patients undergoing surgery for colorectal cancer were administered 200 mg of oral ART daily, in conjunction with a placebo control group. The findings indicated that within the ART group, 67% of patients exhibited > 7% apoptosis of epithelial cells in tumor specimens, versus 55% in the placebo group. Furthermore, Ki-67 serves as a critical prognostic biomarker in colorectal cancer, and artesunate is effective in reducing Ki-67 expression while enhancing CD31 expression. Following two weeks of treatment, the circulating levels of carcinoembryonic antigen (CEA) in the artesunate group were reduced by approximately 75%. During the follow-up period, the recurrence rate was 1 in the ART treatment group, versus 6 recurrences in the placebo group [154].

In a phase I clinical trial involving intravenous ART for patients with advanced solid tumors, including gastrointestinal cancers, 19 patients were enrolled in an accelerated dose-escalation study, with targeted dose levels of 8, 12, 18, 25, 34, and 45 mg/kg, administered on days 1 and 8 of every 21-day cycle. The trial results indicated that the maximum tolerated dose of intravenous artesunate was 18 mg/kg, with four patients demonstrating stable disease, yielding a disease control rate of 27%. Furthermore, dose-limiting toxicities were observed in four patients, encompassing neutropenic fever, hypersensitivity reactions, nausea, and vomiting; however, none of these adverse events were deemed life-threatening [155].

Despite the relatively high safety profile of ART, clinical research into the use of ART for treating gastrointestinal diseases is still relatively limited. Only a limited number of clinical reports on gastrointestinal cancers are available, with almost no research on gastrointestinal inflammation. Nonetheless, ART is regarded as a promising candidate for the treatment of gastrointestinal diseases. The clinical application of ART necessitates robust evidence from extensive clinical trials, as although there are promising results from some case studies, the sample sizes are limited, and ART’s long-term efficacy and safety profiles require further investigation. Furthermore, large-scale clinical trials are required to establish the optimal dosage and treatment protocol for ART in clinical settings, thereby laying the groundwork for its future clinical application in the treatment of gastrointestinal diseases.

## 7. Conclusions

In recent years, the morbidity and mortality of digestive diseases have been increasing, posing a threat to human life and health. Currently, the treatment of digestive system diseases primarily relies on surgical interventions and Western medicine, but these treatments are more harmful to patients and are prone to drug resistance. Numerous studies have demonstrated that the use of Chinese medicine and its integration with Western medicine offer significant advantages, and this is increasingly being recognized as a novel therapeutic approach.

ART has been widely used in clinical practice because of its high efficiency, low toxicity, and low susceptibility to drug resistance. Beyond its substantial therapeutic impact against malaria, ART displays a range of pharmacological properties, including anti-inflammatory, anti-tumor, and anti-fibrotic effects. At present, numerous studies indicate that ART holds promise as a treatment for digestive system disorders, which can resist common digestive system inflammation by inhibiting inflammatory mediator release, alleviating oxidative stress, and promoting ferroptosis in inflamed cells. Furthermore, it can be employed to treat various types of digestive system cancers by inhibiting cell proliferation, invasion, and migration; regulating the progression of the cell division process; and inducing programmed cell death. Nevertheless, gastrointestinal diseases are caused by a complex array of factors, involving multiple elements and signaling pathways, thus presenting a research gap in the understanding of receptors. The mechanism of action by which ART combats gastrointestinal diseases remains unclear. Furthermore, the use of ART succinate in these contexts is currently exploratory and necessitates additional research and clinical validation. Current research is primarily conducted in laboratory environments or small-scale clinical trials and is devoid of support from large-scale, multicenter clinical trial data.

In conclusion, as a compound with significant therapeutic potential, ART, despite the presence of numerous unknowns and challenges at present, warrants active investigation and exploration to establish a foundation for its future clinical utility. Future research should focus on the following key areas: First, an in-depth investigation of the mechanism of action of ART is required to clearly define its targets and signaling pathways in various gastrointestinal diseases, providing a robust theoretical foundation for clinical application. Second, conducting large-scale, multicenter clinical trials is essential to validate ART’s efficacy and safety across a range of diseases. Third, exploring the combination of ART with other drugs to improve therapeutic outcomes while minimizing adverse effects is a critical next step. And fourth, optimizing the formulation process of ART to enhance its bioavailability and stability is vital for its successful clinical implementation.

## Figures and Tables

**Table 1:** Studies of ART in the treatment of gastric ulcers. (“↑” indicates upregulation; “↓” indicates downregulation).

In Vivo/In Vitro	Models/Cells	Machine	Reference
In vivo	Ethanol induction and pyloric ligation to establish a rat gastric ulcer model	GSH↓, SOD↓, TNF-α↓, IL-1β↓, IL-6↓, NF-κB (p65)↓, TBARS↑, MPO↑	[108]
In vivo	Rat model of gastric ulceration induced by aspirin intake	GSH↓, SOD↓, TNF-α↓, IL-1β↓, IL-6↓, NF-κB (p65)↓, COX-2↓, TBARS↑, MPO↑	[109]
In vivo and in vitro	In vitro culture of *Helicobacter pylori* and in vivo experiments with *Helicobacter pylori* PremSS1 strain-infected mice	Inhibit the growth of *Helicobacter pylori*	[110]

**Table 2:** Studies of ART in the treatment of ulcerative colitis. (“↑” indicates upregulation; “↓” indicates downregulation).

In Vivo/In Vitro	Models/Cells	Machine	Reference
In vivo	DSS induces the establishment of mouse models	Bcl-2/Bax↓, cleaved-caspase 3↓, IL-1β↓, IL-6↓, TNF-α mRNA↓, inhibition of p65 and IκBα phosphorylation	[113]
In vivo and in vitro	LPS-induced RAW264.7 cell construction model, TNBS-induced mouse establishment model	miR-155↓, IL-12↓, IL-17↓, IL-23↓, TNF-α mRNA↓, p-NF-κB↑	[114]
In vivo and in vitro	LPS-induced RAW264.7 cell construction model, DSS-induced establishment of rat models	MPO↓, TNF-α↓, IL-8↓, IFN-γ↓, TLR4↓, p-NF-κB↓, Bax↓, caspase-9↓, Bcl-2↑	[115]
In vivo	DSS induces the establishment of mouse models	p-PERK/PERK↓, p-eIF2α/eIF2α↓, p-IRElα/IRElα↓, ATF4↓, XBPls↓	[116]

**Table 3:** Studies of ART in the treatment of hepatic fibrosis. (“↑” indicates upregulation; “↓” indicates downregulation).

In Vivo/In Vitro	Models/Cells	Machine	Reference
In vivo and in vitro	Carbon tetrachloride induces establishment of mouse model, murine-derived hepatic stellate cell line HSC-T6	GPX4↓, Ptgs2↓, Fe^2+^↑, GSH↑, NADPH↓, LPO↑, MDA↑, Atg3↑, Atg5↑, Atg6/beclin1↑, Atg12↑, LC3↑, p62↓, FTH1↓, NCOA4↓	[119]
In vivo and in vitro	Modeling of HF in schistosome-infected mice, LX-2	NDUFB8↓, UQCRC2↓, OCR↓	[120]
In vivo	BSA-induced establishment of rat models	α-SMA↓, MMP-2↓, MMP-9↓, MMP-13↑	[121]
In vitro	LX-2 cells	Bax/Bcl-2↓, α-SMA↓, COL1↓, p-GSK-3β↓, β-catenin↓, p-AKT↓, p-FAK↓	[122]
In vivo	Carbon tetrachloride-induced establishment of SD rat model	TNF-α↓, IL-6↓, α-SMA↓, TLR4↓, MyD88↓, TGF-β1↓, NF-κB p65↓	[123]
In vitro	Rat HSC cells	β-catenin↓, P53 mRNA↓, p53↓	[124]

**Table 4:** Studies of ART in the treatment of gastric cance (“↑” indicates upregulation; “↓” indicates downregulation).

In Vivo/In Vitro	Models/Cells	Machine	Reference
In vitro	SGC-7901cell	CDC25A↓, Bcl-2↓, Bax↑, caspase-3↑	[129]
In vitro	HGC-27cell	COX-2↓, Bcl-2↓, Bax↑, caspase-3↑, caspase-9↑	[130]
In vivo and in vitro	In vitro culturing of SGC7901, BGC-823, and AGS cells and in vivo experiments in nude mouse models	VEGF↓, calpain-2↑	[131]
In vivo and in vitro	In vitro culture of SGC-7901 and GES-1 cells and establishment of *Helicobacter pylori*-induced mouse model	ROS↑, COX-2↓, p-IκBα↓, IκBα↑	[132]

**Table 5:** Studies of ART in the treatment of hepatocellular carcinoma (“↑” indicates upregulation; “↓” indicates downregulation).

In Vivo/In Vitro	Models/Cells	Machine	Reference
In vitro	SK-HEP1, SM7721, HepG2, and Huh7 cell	E-cadherin↓, N-cadherin↓, MMP-9↓, Smad2/3↓	[136]
In vivo and in vitro	HepG2, SNU-182, SNU-449, and Huh7 cells for in vitro experiments, nude mice inoculated with Huh7 cells for in vivo experiments	GSH↓, ROS↑, Cathepsin B/L↑, TFRC↓, FTL↓, FTH↓	[137]
In vivo and in vitro	HCC cell lines, including PLC/PRF/5, HuH7, HepG2, Hep3B, and HCCLM3 cells, nude mice inoculated with Hep3B cells for in vivo experiments	p-ERK↓, p-STAT3↓, Bcl-2↓, Bax↑, caspase-3/7↑	[138]
In vivo and in vitro	HepG2 SR cells for in vitro experiments and inoculation of HepG2 SR cells into mice for in vivo experiments	ROS↑, p-STAT3↓, p-Src ↓, Mcl-1↓, Bcl-2↓	[139]
In vivo and in vitro	SK-hep1 and SM-7721 cells for in vitro experiments and inoculation of SK-hep1 into mice for in vivo experiments	p-AKT↓, p-mTOR↓	[140]
In vivo	Establishment of hepatocellular carcinoma model by in vivo injection of nitroso diethylamine into rats	ALP↓, AST↓, ALT↓, LDH↓, g-GT↓, IL-6↓, JAK-2↓, STAT-3(pY705)↓, Bcl-xL↓, Bcl-2↓, caspase-3↑	[141]
In vivo and in vitro	In vitro experiments with HepG2 and MHCC-97H cells and establishment of rat model with HepG2 cells	LC3B↑, SQSTM1/p62↑, GBA↓	[142]

**Table 6:** Studies of ART in the treatment of colorectal cancer (“↑” indicates upregulation; “↓” indicates downregulation).

In Vivo/In Vitro	Models/Cells	Machine	Reference
In vivo and in vitro	DLD-1, HCT116, HCT15, and SW480 cells were used for in vitro experiments, and mouse model of intestinal polyps was established for in vivo experiments	c-Myc↓, cyclinD1↓, TCF1/TCF7↓	[145]
In vivo and in vitro	HCT116, HT29, SW480, SW620, COLO325, COLO205, HCT15, and RKO cells; mouse models were established with HCT116 cell inoculation	KRAS↓, ANACP-2↑, βTrCP↑, GSK-3β↑, AKT↓, PI3K↓, mTOR↓	[146]
In vitro	SW480 and HCT116	ROS↑, CDK2/4/6↓, p16↑, p21↑, LC3B↑, p62↑, Beclin1↑, Atg3↑, Atg5↑, Atg7↑, Atg12↑, BIP↓, p-IRE1α↓, IL-6↑, MMP-3↑	[147]
In vivo	Establishment of rat model of colorectal cancer induced by 1,2-dimethylhydrazine	GSH↑, SOD↑, TBARS↓, MPO↓, TNF-α↓, PGE_2_↓, COX-2↓, iNOS↓, NF-κB↓, IL-1β↓	[148]
In vivo and in vitro	HCT116 cells, colorectal cancer modeling with HCT116 cells	caspase-3↑, Bax↑, PARP↑, caspase-9↑, Bcl-2↓, Bcl-xL↓, beclin-1↑, LC3-I/II↑, Atg5↑, Atg12-Atg5↑	[149]

## Abbreviations

ART	Artesunate
DHA	Dihydroartemisinin
C_max_	Peak concentration
t_1/2_	Elimination half-life
T_max_	Time to maximum concentration
CYP450	Cytochrome P450
DDP	Cisplatin
ADM	doxorubicin
PTX	Paclitaxel
JAK	Janus kinase
STAT	Signal transducer and activator of transcription
ABC	ATP-binding cassette
ABCG2	ATP-binding cassette subfamily G member 2
TLR4	Toll-like receptor 4
NF-κB	Nuclear factor kappa B
IFN-γ	Interferon-γ
IL	Interleukin
TNF-α	Tumor necrosis factor-α
ERK	Extracellular regulated protein kinase
NLRP3	Nucleotide-binding oligomerization domain leucine-rich repeat and pyrin domain-containing 3
AMPK	Adenosine 5′-monophosphate (AMP)-activated protein kinase
mTORC1	Mechanistic target of rapamycin complex 1
GPX4	Glutathione Peroxidase 4
mTOR	Mammalian Target of Rapamycin
PI3K	Phosphatidylinositol 3 kinase
AKT	Protein kinase B
Wnt	Wingless
ATF4	Activating transcription factor 4
CHOP	C/EBP homologous protein
CHAC1	ChaC Glutathione-Specific Gamma-Glutamylcyclotransferase 1
ULK1	Unc-51-like kinase 1
TGF-β1	Transforming growth factor-β 1
Smad2/3	Recombinant Mothers Against Decapentaplegic Homolog 2/3
LPS	Lipopolysaccharide
PDE4D	Recombinant Phosphodiesterase 4D
cAMP	Cyclic adenosine monophosphate
MAPK	Mitogen-activated protein kinase
GSDMD	Gasdermin D
GDF15	Growth differentiation factor 15
GFRAL	GDNF family receptor alpha-like
RAGE	Receptor for advanced glycosylation end products
pH	Pondus Hydrogenii
GSH	Glutathione
SOD	Superoxide dismutase
TBARS	Thiobarbituric acid reactive substances
MPO	Myeloperoxidase
ADEF	Artesunate dry emulsion formulation
*H. pylori*	*Helicobacter pylori*
PPIs	Proton pump inhibitors
COX-2	Cyclooxygenase-2
UC	Ulcerative colitis
DSS	Dextran sulfate sodium
Muc2	Mucin 2
Bcl-2	B-cell lymphoma-2
Bax	Bcl-2-associated X protein
IκBα	Inhibitor of NF-κB α
GRP78	Glucose-regulated protein 78 kD
p-PERK	Phospho-PERK
eIF2α	α subunit of eukaryotic initiation factor 2
ATF4	Activating transcription factor 4
CHOP	C/EBP homologous protein
p-eIF2α	Phosphorylated α subunit of eukaryotic initiation factor 2
IRE1α	Inositol-requiring enzyme-1α
IRE1α	Phospho-IRE1α
XBP1	X-Box Binding Protein 1
TNBS	Trinitro-benzene-sulfonic acid
miR-155	MicroRNA 155
HF	Hepatic fibrosis
ECM	Extracellular matrix
HSCs	Hepatic stellate cells
GPX4	Glutathione Peroxidase 4
Ptgs2	Prostaglandin-endoperoxide synthase 2
LPO	Lipid hydroperoxide
MDA	Malondialdehyde
ROS	Reactive oxygen species
NADPH	Nicotinamide adenine dinucleotide phosphate
LC3	Microtubule-associated protein 1 light chain 3
FTH1	Ferritin heavy chain 1
NCOA4	Nuclear receptor coactivator 4
*Atg*	Autophagy-related gene
OCR	Oxygen consumption rate
MMP	Matrix metalloproteinase
α-SMA	Alpha smooth muscle Actin
COL1	Collagen Type I
GSK-3β	Glycogen Synthase Kinase-3β
FAK	Focal Adhesion Kinase
MyD88	Myeloid Differentiation Factor
CDC25A	Cell division cycle 25 homolog A
VEGF	Vascular Endothelial Growth Factor
TFRC	Vascular Endothelial Growth Factor
FTL	Ferritin light chain
ALP	Alkaline Phosphatase
AST	Aspartate Aminotransferase
ALT	Alanine Aminotransferase
KRAS	Kirsten rats arcomaviral oncogene homolog
CEA	Carcinoembryonic antigen
